# Unmasking inborn errors of immunity: identifying the red flags of immune dysregulation

**DOI:** 10.3389/fimmu.2024.1497921

**Published:** 2024-12-19

**Authors:** Manuela Cortesi, Laura Dotta, Marco Cattalini, Vassilios Lougaris, Annarosa Soresina, Raffaele Badolato

**Affiliations:** Pediatrics Clinic and Institute for Molecular Medicine “A. Nocivelli”, Department of Clinical and Experimental Sciences, University of Brescia and ASST-Spedali Civili di Brescia, Brescia, Italy

**Keywords:** inborn errors of immunity, immune dysregulation, autoimmunity, APDs, warning signs

## Abstract

Inborn errors of immunity (IEI) are rare diseases that affect the immune system. According to the latest International Union of Immunological Societies (IUIS) classification, 485 different IEI have been identified. Even if increased susceptibility to infections is the best-known symptom, IEI are no longer defined by the higher likelihood of infections alone. Immune dysregulation with autoimmune disease and hyperinflammation, lymphoproliferation, and malignancy are common manifestations and could be the only symptoms of IEI that must be recognized. An exclusive focus on infection-centered warning signs would miss around 25% of patients with IEI who initially present with other manifestations. Timely and appropriate diagnosis and treatment are essential to enhance the quality of life (QoL) and, in some cases, survival, as patients are susceptible to life-threatening infections or autoimmunity. In addition, the advantage of early diagnosis in IEI with immune dysregulation (i.e. *CTLA4* deficiency, *LRBA* deficiency, *NF-kB1/NF-kB2* deficiency, activated phosphoinositide 3-kinase delta syndrome -APDS-) is the initiation of targeted therapies with precise re-balancing of the dysregulated immune pathways (i.e., biologicals, selective inhibitors) or definitive therapy (i.e., HSCT).

## Introduction

The group of Inborn Errors of Immunity (IEI) currently comprises 485 disorders of immune function and/or regulation, most of them with a specific monogenetic cause (1). These conditions are classified into 10 categories according to their clinical and immunologic phenotypes by the International Union of Immunological Societies (IUIS) (1).

IEI are inherited immune system disorders with a broad spectrum of manifestations, starting with an increased susceptibility to infections, but also often including immune dysregulation and malignancy. Earlier diagnosis helps ensure that the patient is prescribed the most appropriate therapeutic intervention, improving prognosis (2) and decreasing the risk of infection and inappropriate vaccinations (3, 4).

Historically, IEI were defined as pathologies resulting in increased susceptibility to severe or recurrent infections, and was known as primary immunodeficiencies (PID). In the last 30 years, the understanding of what comprises a PID has significantly expanded, and to recognize this wider spectrum manifestations, the term inborn error of immunity has been introduced for these conditions, replacing the term PID in the 2019 classification by the International Union of Immunological Societies (5).

Recent discoveries in the field of IEI enabled the better characterization of the non-infectious manifestations of IEI, specifically immune dysregulation.

Immune dysregulation, which is autoimmune or autoinflammatory manifestations, is defined as at least 1 of the following: chronic lymphoproliferation (hepatomegaly, splenomegaly, lymphadenopathy); autoimmunity (i.e. cytopenia, hepatitis, autoimmune arthritis and other rheumatologic diseases, thyroid disease, vitiligo, alopecia, diabetes; celiac disease); granuloma; inflammatory bowel disease (IBD) or enteropathy; severe allergies; or autoinflammatory symptoms (i.e. persistent fever, rashes, elevated inflammatory markers) (5).

In some patients autoimmune or autoinflammatory may be the only indicators of IEIs (5). These cases may be categorized as “diseases of immune dysregulation” according to the most recent classification from the International Union of Immunological Societies (IUIS) 2022 (1). Other types of IEIs include immune dysregulation as part of a wider clinical phenotype (6).

The latest IUIS lists “Diseases of immune dysregulation” as an independent category of IEI (1), including familial haemophagocytic lymphohistiocytosis (FHL syndromes), FHL syndromes with hypopigmentation, regulatory T cell defects, autoimmunity with or without lymphoproliferation, immune dysregulation with colitis, autoimmune lymphoproliferative syndrome (ALPS, Canale-Smith syndrome) and susceptibility to EBV and lymphoproliferative conditions.

Patients with these diseases may suffer from immune dysregulation alone, or with a combined manifestation of immune deficiency, autoimmunity, recurrent inflammation, lymphoproliferation and even predisposition to malignancy.

**Figure 1:**
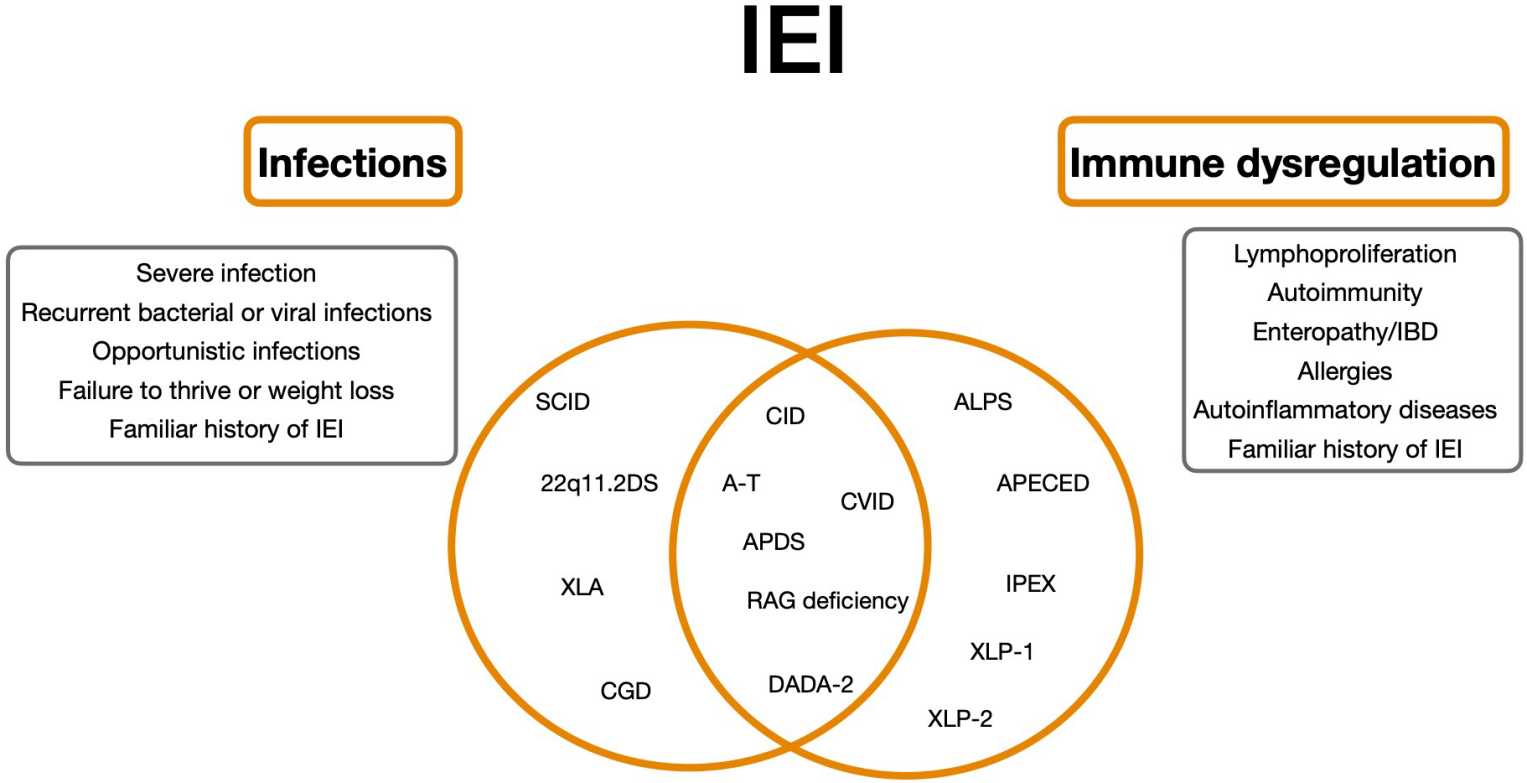
Clinical spectrum of Inborn Errors of Immunity as a continuum. 22q11.2DS, 22q11.2 deletion syndrome; ALPS, autoimmune lymphoproliferative syndrome; APDS, Activated Phosphoinositide 3-kinase-d-syndrome; CVID, Common variable immunodeficiency; SCID, Severe combined immunodeficiency; CID, Combined Immunodeficiency; XLA, X-linked agammaglobulinemia; A-T, Ataxia-teleangiectasia; DADA-2, deficiency of adenosine deaminase 2; IPEX, immune dysregulation, polyendocrinopathy, enteropathy, X-linked stndrome; XLP-1/2, SAP e XIAP deficiency.

However, overlap between immunodeficiency and autoimmunity is commonly observed in patients with other various IEI (7) (**Figure 1**).

Mechanisms preventing adequate response against pathogens also affect self-tolerance and immune regulation (8).

As described by Fischer et al. (9) immune dysregulation is associated with virtually all IEI and is observed in a significant group of patients with IEI, with a wide range of manifestations. These manifestations occurred throughout the patient’s lifetime and have a prognostic significance.

Immunoregulatory disorders are at least 10 times more frequent in patients with IEI than in the general population (9). This increase in risk is extremely high for autoimmune cytopenia (by a factor of 120), but also with the 80-fold increased risk of inflammatory bowel disease, and the 40-fold increased risk of arthritis (9), suggesting that patients presenting with immune dysregulation should be screened for IEI, ideally, but perhaps not exclusively, during childhood.

Although autoimmune manifestations occurred in patients with all types of IEI and are a significant component of the clinical presentation of all types of IEI, those with T-cell defects or common variable immunodeficiency (CVID) tended to have the highest risk for autoimmunity (10).

As described by Thalhammer et al., even if infection is the most frequent initial presenting manifestation of IEI, 18% of patients present with immune dysregulation as the first symptom (5).

## Demographics

Individual IEI disorders are rare, but IEI as a collective account for a significant group of diseases, affecting around 1 in 2000 people (11–13).

When reviewing patients affected by IEI, after excluding patients with an isolated haemophagocytic lymphohistiocytic syndrome or an IEI characterized by autoimmunity and lymphoproliferation in the absence of significant infections^1^, the risk of autoimmunity/inflammation was 24.6% and thus did not show any great difference from the overall study population (9). It is worth noting that all groups of patients classified as having CVIDs, other B-cell IEIs, T-cell IEI, or innate immune system deficiencies were at risk of autoimmune or inflammatory manifestations. Those patients at highest risk were those with CVID and combined immunodeficiencies and 25% of patients with innate IEI had autoimmune or inflammatory manifestations (9). These manifestations occurred earlier in life in patients with T- cell and innate immune deficiencies than in patients with B-cell IEI. In contrast, cytopenia and rheumatologic manifestations were more frequently associated with T-cell IEI than other IEI (9).

Considering the first presenting symptoms in relation to the age of onset, at early onset age (6-25 years) immune dysregulation was the initial presentation of IEI in approximately 25% of patients, associated or not with infection. However, beyond age 30 infection without immune dysregulation was the dominant initial presentation in around 80% of patients with IEI (5).

Analysis of the ESID registry indicates there are more male than female patients (56 vs 44% respectively), suggested to be explained by the X-linked inheritance of many IEI. This, plus the early onset before age 6 leads to a particular predominance in males within the first years of life which predictably shifts to a female predominance with increasing age, most evident after 50 years (5).

There is also a significant change observed in the sex ratio of patients presenting with immune dysregulation with increasing age. In patients under 10 years of age, more than 60% with immune dysregulation as only or concomitant initial manifestation were boys, whereas male/female ratios were similar between age 10-30 years. It is only beyond 30 that females predominated, reaching up to 70% after age 40 (5).

Considering all these data, there is growing awareness that autoimmune disorders may precede infections among patients with IEI, especially those with primary immune regulatory diseases (PIRD) (5). Therefore, initiatives are critical to expedite a diagnosis and repurpose mechanism-based biologics for targeted therapies (14).

Immune dysregulation accounts for a large proportion of manifestations in IEI patients, resulting in a worse prognosis in patients with immune dysregulation compared to those with only high infection susceptibility (9).

Among immune dysregulation symptoms, autoimmune cytopenias are often dominant in the initial clinical presentation, but patients may eventually progress to autoimmune enteropathy, lymphoproliferation, rheumatologic diseases, and autoinflammatory disorders.

### Autoimmune cytopenias

Cytopenias can be the presenting symptom of many IEI, and are defined as the reduction of one or more mature blood cell types in the peripheral blood. Autoimmune cytopenias (AICs) are acquired conditions caused by immune-mediated destruction of mature peripheral blood cells (15), including immune thrombocytopenic purpura (ITP), autoimmune haemolytic anaemia (AIHA), autoimmune neutropenia (AIN), and Evans syndrome (ES). They are common presentations of IEI in the pediatric age group, with at least 65% of cases of ES being genetically determined (16).

ITP and AHIA often present as the first symptom in adults too. Most children with AHIA, ITP, and AIN have a mild to moderate clinical course of the disease, often requiring only observation and treatment with first-line therapies (17).

In general, if a patient does not respond to first-line therapy, a diagnostic re-evaluation is needed, focusing on causes of secondary AICs.

Currently, there are no specific diagnostic biomarkers to identify the patients at risk of IEI among those presenting with autoimmune cytopenia, but unusual age at disease onset (early chronic immune thrombocytopenia, late onset of autoimmune neutropenia), positive family history for IEI or immune dysregulation, chronic disease course, and refractory disease should be considered as elements of interest. In these cases, immunological investigations are always recommended (18).

The identification process for IEI includes a first line assessment including the dosage of serum immunoglobulin levels (IgA, IgM, IgG, and IgE), the evaluation of anti-vaccine antibody response and extended immunophenotype. If these parameters are altered or in the presence of clinical risk indicators for IEI, it is mandatory to proceed with second level investigations such as proliferation tests in response to mitogens along with various molecular tools (15). There are a number of specific immunological alterations including humoral and cell-mediated immune defects, that are red flags for an associated IEI if accompanied by AIHA, ITP, AIN or ES, for example reduced serum immunoglobulin and low T-cell counts. However, in a number of patients with suspected IEI, immunological tests can be normal (19, 20).

**Figure 2:**
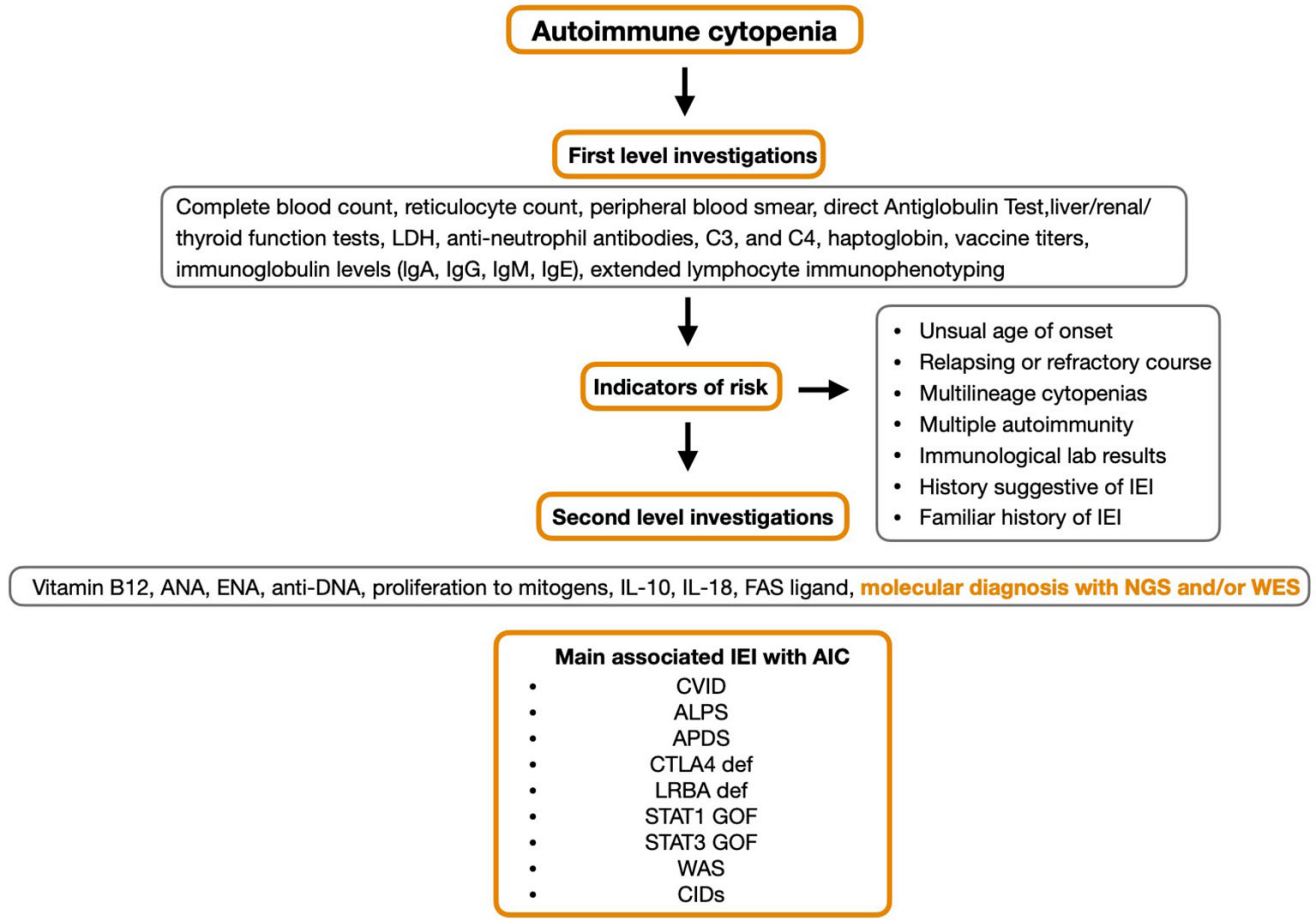
Critical approach to patients with autoimmune cytopenias.

In AIC patients with IEI, cytopenias affecting multiple blood lineages or those associated with adenopathy and/or hepatomegaly/splenomegaly or corresponding infections are characteristic traits (21) (**Figure 2**).

AIC patients with IEI often do not respond to first-line therapy and further investigation into best practice for these patients still needs to be understood (18). In addition, the ability to obtain a definitive molecular diagnosis may present new opportunities for targeted therapeutic options as seen in *LRBA* and *CTLA-4* deficiencies.

### Non-malignant pediatric lymphoproliferative disorders

One specific class of disorders, non-malignant pediatric lymphoproliferative disorders (PLPD) which can be characterized by the presence of proliferating clonal or polyclonal lymphoid cells, can occur in response to immune stimuli and can indicate underlying immune dysregulation (15).

Clinical presentations include chronic (>3 months) or recurrent lymphadenopathy, splenomegaly, or symptoms secondary to organ infiltration by lymphoid cells.

PLPD has also been noted to be linked to an increased incidence of lymphoma and other haematopoetic malignancies (22).

Lymphadenopathy can create diagnostic challenges when it is the first presenting symptom of an immune system disorder, particularly in children, resulting in delayed diagnosis (22).

Lymphadenopathy is common during childhood and it poses a significant diagnostic dilemma when it represents the first sign of a disorder of the immune system, leading to a consequently delayed diagnosis. While transient lymphadenopathy in children is often benign, long-standing lymphoproliferation may reflect underlying immune dysregulation, increase the risk for developing malignant disease, and/or drive life-threatening lymphoproliferative disease (23).

When lymphadenopathy is identified in patients who have been diagnosed with immunodeficiency, it should be considered a red flag to consider differential diagnosis between benign lymphoproliferation and malignancy (24).

Lymphadenopathy as well as hepatosplenomegaly can be part of the clinical spectrum of several IEI, including diseases with immune dysregulation and autoinflammatory disorders, as the clinical expression of benign polyclonal lymphoproliferation, granulomatous disease or lymphoid malignancy (24).

Although several inherited diseases of immune dysregulation have been associated with PLPD, frequency and distribution of PLPD in children with IEIs are unknown.

**Figure 3:**
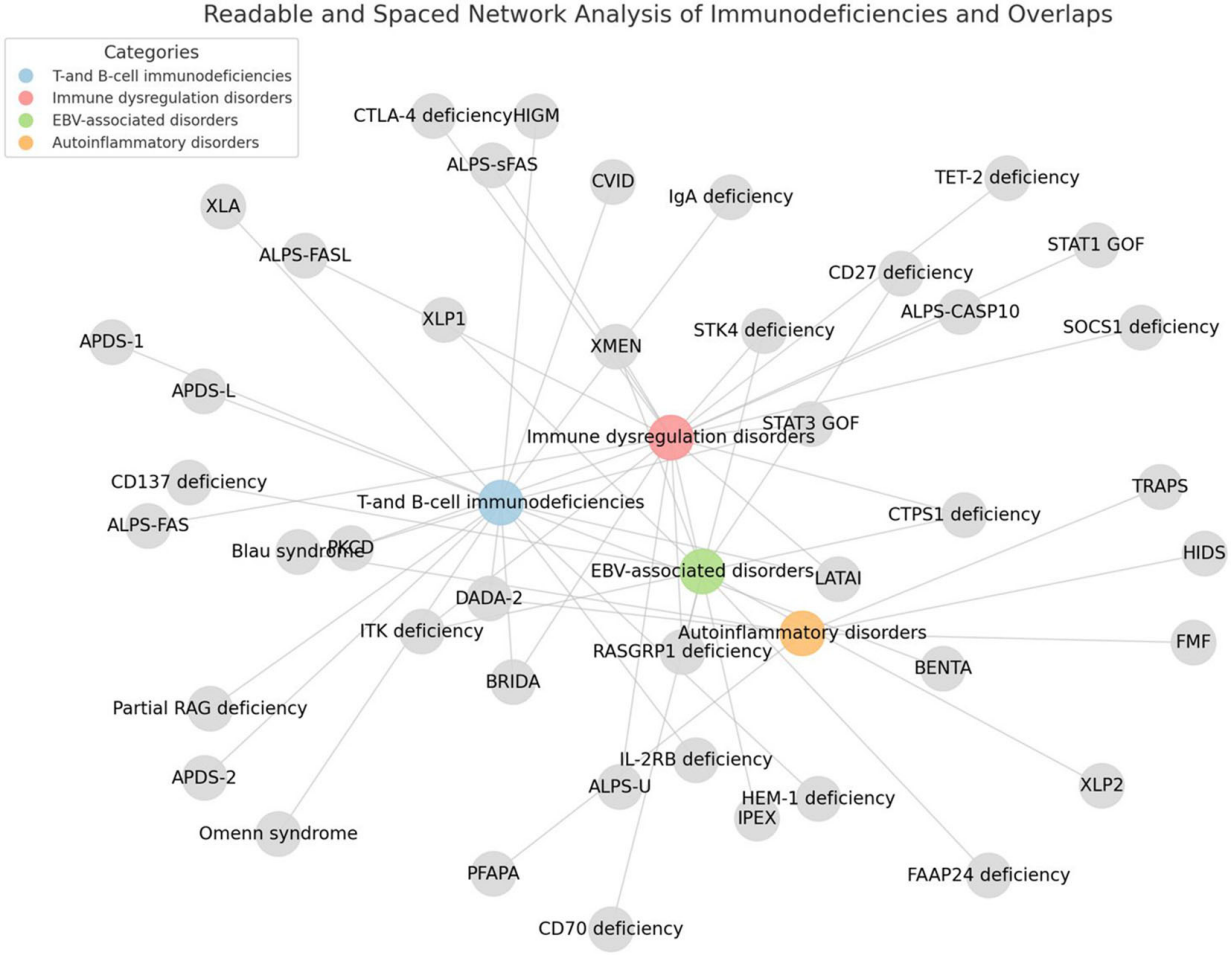
Inborn errors of immunity associated with non-malignant pediatric lymphoproliferative disorders. The figure was generated using Python and the NetworkX library.

Errors in more than 400 genes are now ascribed to IEI and a significant number of these conditions present with clinical features consistent with PLPD (22) (**Figure 3**).

The mechanisms underlying lymphoproliferation in the different immune system disorders are multiple and have yet to be completely understood.

However, recent advances in genetic techniques will allow for increased opportunities to identify new disorders, which in turn will allow genotype-phenotype connections to be made, resulting in improved treatment and follow-up. Forbes et al., noted the identification of pathogenic gene variants implicated in PLPD were able to allow use of targeted therapies and to reduce mortality (22).

### Rheumatologic and other autoimmune diseases

The signs and symptoms of most rheumatic diseases are classified in international ACR or EULAR criteria. Rheumatologic disorders are considered complex diseases with partial genetic origin, with a heterogeneous genetic background and variable phenotypic presentation. An increase in research into the genetics behind these diseases has led to the detection of several genetic mutations that are linked to the immune dysregulation seen in many rheumatic diseases.

In recent years, the increased interest in identification of single-gene causes of the rheumatologic diseases, has resulted in identification of several genetic mutations that underlie immune dysregulation and subsequently rheumatological manifestation (25).

A high degree of overlap exists between autoimmune diseases and inborn errors of immunity in terms of genetic associations and causes (7).

There is a significant increase in autoimmune and autoinflammatory complications in IEI patients, especially in common variable immunodeficiency (CVID) and combined immunodeficiency (CID). Patients with these complications have a shorter life expectancy or increased mortality (9).

From a genetic point of view, rheumatoid arthritis (RA) and systemic lupus erythematosus (SLE) are the two best-studied rheumatic diseases. Different genes associated with IEI and involved in immune regulation are relevant to rheumatic diseases (**Table 1**).

**Table 1:** Inborn errors of immunity resulting in rheumatologic disease.

IUIS category	Gene(s)	Pathogenesis	Phenotype
IEIs affecting cellular and humoral immunity	RAG1/2 ZAP70 DCLRE1 LCK PRKCD TAP1/2 IL2RG STK4 ADA CD3G OX40 LAT CD40L	T cell dysfunction	Arthritis, vasculitis, panniculitis, alopecia, SLE/SLE-like disease
Predominantly antibody deficiencies	BTK ICOS TNFRSF1B TNFRSF1C IKAROS NFKB1 NFKB2 CVIDs w/o genetic cause	B cell dysfunction	Arthritis, vasculitis, panniculitis, alopecia, Sjögren syndrome
Disease of immune dysregulation	FOXP3 CD25 WAS ARPC1B CTLA4 LRBA PIK3CD PIK3R1 STAT3 GOF STAT1 GOF	Loss of peripheral immune tolerance	Arthritis, vasculitis, Sjögren syndrome, SLE/SLE-like disease
Disease of immune dysregulation	AIRE	Loss of central immune tolerance	Arthritis, alopecia
Congenital defects of phagocyte number and function	CYBB CYBA NCF1 NCF2 NCF4 EROS	Defects of phagocytosis	Arthritis, vasculitis, Sjögren syndrome, SLE/SLE-like disease
Complement deficiencies	C1QA C1QB C1QC C1R C1S C4A/B C2 C3 C5 C6 C7 C8A C8G C8B C9 MASP2 CFP CFI CFHR1-5 THBD FCN3 SERPING1 CFB CFD	Impaired clearance of immune complexes	Arthritis, vasculitis, Sjögren syndrome, SLE/SLE-like disease

SLE, systemic lupus erythematosus.

Rheumatologic diseases that present with conditions in multiple systemic pathways, in combination with resistance to therapy, may be indicative of a possible IEI. In addition, cases of SLE or early onset connective tissue disease (particulary before 5 years of age), consanguinity or endogamy (because some defects are of autosomal recessive inheritance), familial cases of autoimmunity (because some defects are of autosomal dominant inheritance), and severe or refractory disease may also be indicators to consider IEI as the root (26).

In general, rheumatologic diseases presenting within a multisystem disease spectrum as well as therapy resistance may alert clinicians considering IEI as a possible diagnosis.

Furthermore, the case of early-onset connective tissue disease, and particularly SLE, should raise the suspicion of a potentially underlying IEI.

## Autoimmune endocrinopathies

The association between endocrine autoimmune diseases and IEI is currently well known. However, when endocrinopathies represent the main symptom or develop before the patient has experienced recurrent infections, an underlying IEI may be missed and its diagnosis delayed for years.

Clinical clues that a patient with endocrinopathy may have an underlying IEI are early disease onset, the development of autoimmune processes affecting multiple organ systems, not necessarily at the same time, and the positive familial history for endocrine disorders and autoimmunity.

Autoimmune thyroid diseases or other endocrinopathies, such as type 1 diabetes mellitus, primary hypoparathyroidism, and Addison disease, are seen in IEI.

Even though autoimmune polyendocrine syndrome 1 (APS-1) represents the paradigm of the immune disorder associated with endocrinopathy, other disorders such as immune dysregulation, polyendocrinopathy, enteropathy, X-linked (IPEX) syndrome, *CTLA4* deficiency, *LRBA* deficiency, *STAT1* gain of function (GOF), *NFKB2* LOF, *STAT3* GOF must be considered.

### Inflammatory bowel disease

Inflammatory bowel diseases (IBD) are chronic inflammatory conditions of the gastrointestinal tract, including Crohn’s disease, ulcerative colitis (UC) and inflammatory bowel disease-undefined (IBD-U). IBD are understood to be multifactorial, involving genetic, immune, microbial and environmental factors. Advances in next generation sequencing promoted the identification of over 80 monogenic causes of IBD, many of which overlap with IEI. Approximately one third of currently identified IEI result in gastrointestinal manifestations, most being inflammatory in nature, such as IBD (27). IEI resulting in monogenic IBD are disorders involving the intestinal epithelial barrier, phagocytosis, T and B cell defects, central and peripheral tolerance.

Consequences of the breakdown of the immune system are strongly reflected in the gastrointestinal tract and often result in disease at an especially young age.

Monogenic IBD is predominantly identified in patients diagnosed prior to 6 years old, known as very early onset IBD (VEO-IBD) (28) (**Table 2**).

**Table 2:** Monogenic causes of IBD that are concurrent IEI, defined according to IUIS 2022.

IUIS category	Gene(s)
IEIs affecting cellular and humoral immunity	ADA RAG1 RAG2 IL2RG DCLREIC LIG4 ZAP70 CD3G DOCK8 CD40LG ICOS MALT1
CID with associated or syndromic features	WAS ARPC1B IKBKG TGFBR1 TGFBR2 TTC7A ORAI1 STIM1 TTC37 SKIV2L ZBTB24
Predominantly antibody deficiencies	BTK PIK3CD PIK3R1 PTEN TRNT1 AICDA
Disease of immune dysregulation	SH2DIA XIAP CASP8 ITCH FOXP3 LRBA STAT3-GOF FERMT1 CTLA4 IL10R IL10RA IL10RB RIPK1 TGFB1 IL21 IL2RA IL2RB
Congenital defects of phagocyte number, function, or both	G6PC3 ITGB2 CYBB CYBA NCF1 NCF2 NCF4
Defects in intrinsic and innate immunity	STAT1 GOF
Auto-inflammatory disorders	MEFV MVK PLCG2 NLRC4 POLA1 TRIM22 TNFAIP3
Complement deficiencies	CD55 MASP2 FCN3
Bone marrow failure	DKC1 RTEL1

Among patients with monogenic IBD, employing conventional therapeutics is oftentimes inadequate. Indeed, biologics are only effective in 25.5% of patients with monogenic IBD (28).

Moreover, among VEO-IBD patients, monogenic disease is a driver of disease severity, including death (29). Given the growing number of IEI that occur with IBD, it is necessary, especially in front of a VEO-IBD, to exclude an underlying monogenic cause. Understanding the mechanisms driving intestinal inflammation reveals pathways of importance that merit evaluation for therapeutic targeting, facilitating personalized therapeutic options and optimizing prognosis.

### Allergic diseases

Allergy develops on account of disturbed function of the immune system. The immune system depends on a complex balance of activation, to defend against invasive, foreign pathogens, and control, to differentiate between self and non-self.

Allergic manifestations depend on exaggerated immune responses against specific non-self-antigens, known as allergens (30). Frequent allergic symptoms include eczema, allergic rhinitis, asthma, and food allergy, associated with increased serum immunoglobulin (Ig) E and peripheral blood eosinophilia. It is now known that allergic manifestations can be the main symptoms of IEI (31, 32) (**Table 3**).

**Table 3:** Six different phenotypes of IEI associated with allergies.

Clinical phenotypes	Genes
Hyper-IgE syndromes(HIES)	STAT3 DOCK8 ZNF341 IL6ST IL6R PGM3 ERBB2IP TGFBR1 TGFBR2 SPINK5 TYK2
Omenn syndrome	RAG1 RAG2 IL2RG IL7R LIG4 ADA DCLRE1C RMRP CHD7 ZAP70 22q11del
Wiskott-Aldrich syndrome (WAS) and WAS-like conditions	WAS WIPF1 ARPC1B CDC42
Immunodysregulation, polyendocrinopathy, enteropathy, X-linked (IPEX) and IPEX-like conditions	FOXP3 IL2RA STAT5B LOF STAT1 GOF ITCH
CBM-opathies	CARD11 LOF CARD14 MALT1
Other IEI presenting with atopic phenotypes	Selective IgA defiency CARMIL2 JAK1 MYD88 IKBKG NFKB1 NFKB2 STAT6 STAT5B- somatic mutations

CMB, caspase recruitment domain family member 11 (*CARD11* or *CARMA1*)—B cell CLL/lymphoma 10 (*BCL10*)—*MALT1* paracaspase (*MALT1*).

In particular, the allergic triad defined by increased IgE, eosinophilia, and eczema is shared by different IEI that may be misdiagnosed as common allergic diseases (32).

Patients affected by IEI with atopic phenotypes usually present with distinguishing associated clinical manifestations and laboratory findings that need to be promptly identified. In addition, it is fundamental to assess the presence or absence of a familiarity for IEI and/or consanguinity.

Common features of IEI associated with atopic phenotypes are:

1. Early-onset atopic disease, usually at birth or in the first months of life.
2. Severe and recurrent atopic disease, usually not responsive to standard therapy.
3. High levels of Th2 biomarkers (i.e. increased total serum IgE, eosinophilia).
4. Presence of other affected family members, family history of consanguinity.
5. Associated skeletal features.
6. Associated susceptibility to fungal or bacterial infections.
7. Efficacy of targeted therapies.

In general, red flags of IEI that the clinician should recognize are the presence of neonatal erythroderma, congenital ichthyosis, serum total IgE >2000 kU/L, especially in the first 3 months of life, and atopic manifestations associated with recurrent/severe bacterial or viral infections, autoimmunity, cytopenias, chronic diarrhea and/or failure to thrive (30).

### Diseases with granulomatous manifestations

Granulomas are inflammatory infiltrates, which can occur in different tissues as a self-limited or severe condition that can persist for years. Granulomas represent the result of an immune response induced by an encounter between antigen presenting cells (predominantly monocytes and macrophages) and T cells (both CD4 + and CD8+ T cells) (33).

Granulomas can be associated with infectious or inflammatory diseases, and it is hypothesized that they occur as a result of antigen persistence or immune dysregulation. Granulomas are more common in adults than children with IEI (34), and non-infectious granulomatous inflammation is common patients with chronic granulomatous disease (CGD), ataxia telangiectasia (AT), CVID, and severe combined immunodeficiency (SCID) (35, 36).

### Neurological manifestations

The spectrum of manifestations of IEI is continually expanding. Among the various presenting symptoms of these diseases, we must not forget the neurological manifestations.

Although central nervous system involvement and vasculopathies are present only in a minority of patients, systemic and central nervous system vasculitis and also vascular malformations and MoyaMoya syndrome, have recently been reported as presenting or accompanying symptoms in some IEI.

In patients with *CTLA-4* deficiency, for example, neurological involvement is present in almost 30% of cases, ranging from forms of autoimmune encephalitis to inflammatory disorders, optic neuritis and lymphoproliferative diseases with infiltration of the central nervous system.

Neurological involvement in *CTLA-4* deficiency represents the presenting symptom in approximately 5% of patients.

The identification of this form of congenital defect of immunity underlying the neurological manifestations also justifies the use of target therapy with abatacept which was also effective in controlling neurological symptoms (37).

The involvement of the central nervous system may be present, in the form of vascular alterations (aneurysms, vascular ectasias, thrombotic events) also in Hyper-IgE Syndromes.

In those caused by loss of function mutation of *DOCK8*, nervous involvement most often occurs in form of CNS vasculitis and Moyamoya syndrome (38).

Neurological involvement is also present in up to 75% of cases in *DADA2* deficiency. Neurological manifestations can be an initial symptom of the disease and can recur, typically presenting in the form of ischemic strokes, but also as central or peripheral neuropathy (37).

Renal, intestinal, pulmonary, but also cerebral vasculitis is a well-known, and sometimes fatal, complication of Wiskott-Aldrich Syndrome (39).

The literature lacks sufficient evidence regarding vasculitis of the central nervous system associated with IEI and shared consensus regarding treatment. However, the identification of a causative immune defect allow, in selected cases, the initiation of target therapy.

Furthermore, an important consideration regarding the treatment of patients with CNS vasculitis is the risk they have of developing stroke due to inflammation and stenosis of the involved vessels. Recognizing these symptoms as early as possible allows aggressive management of the initial inflammation, in order to control the progression of the disease.

## Diagnostic approach in patients with immune dysregulation

In recent years, there has been a rapid growth of IEI discoveries and a deeper understanding of their mechanisms. It has come to our knowledge that IEI does not only mean higher susceptibility to infections. Those patients with a combination of several types of autoimmune or inflammatory diseases should be considered for a scan of IEI.

Identifying an underlying IEI in a heterogeneous group of patients with a variety of autoimmune disorders requires an immune evaluation in the initial workup to get a specific diagnosis of highly vulnerable patients with genetic immune deficiency disease (10).

The modern diagnostics of IEI include various diagnostic measures, such as a simple blood count with particular attention paid to the total absolute lymphocyte count, the serum immunoglobulin levels, and the complete sequencing of the exome or genome (40).

Current guidelines recommend performing advanced immunological diagnostics of suspected IEI patients before molecular testing (41).

Genetic sequencing is the current standard for confirming the presence of IEI. This is done via predefined panels of sequenced genes or whole exome sequencing (WES). In addition, whole genome sequencing (WGS) can be performed in addition to, or in replacement of a panel or exome approach (42).

As individually diagnosed IEI are considered rare, it is challenging to conduct large-scale studies and thus it is difficult to treat such conditions with limited available data. The advancements in WES and WDS approaches have helped to improve the identification of the causative mutations for IEI, resulting in a doubling of identified mutations between 2009-2019 (43).

## Moving forward: from recognition to intervention

Early detection of genetic diagnoses in immune dysregulatory disorders informs mechanisms of pathogenesis, facilitates assessments of clinical risks, and allows potential therapeutic targets and/or definitive HSCT, potentially avoiding the morbidity and mortality associated with uncontrolled disease and broad immunosuppression.

Therapeutic strategies for IEI affected patients are mainly symptomatic, with the goal of avoiding complications using antibiotics, immunoglobulin replacement, corticosteroids, and immunosuppressive agents, depending on the underlying IEI (17).

Control of immune dysregulation with immunosuppressive therapies should be balanced against the increased risk of infection posed by the underlying defect and accumulated end-organ damage (44).

Nonetheless, understanding the molecular basis of IEI disorders enables the physicians to exploit the molecular defect with targeted therapies.

An improved understanding of the pathophysiology of these disorders has led to the development of mechanism-based therapeutic strategies. Small molecules and biologics are effective in reversing clinical manifestations of primary immune deficiencies and as a bridge treatment to hematopoietic stem cell transplantation (45) (**Table 4**).

**Table 4:** Therapeutic strategies in IEI associated with immune dysregulation.

Disease	Therapeutic options	Targeted therapy	Other therapies
SCID	//	//	HHSCT, gene therapy
CVID, XLA, HIGM	IVIG, Immunosuppression	Rituximab	Splenectomy
APS-1	-	Ruxolitinib	//
XLP-1	IVIG	Rituximab	HSCT
ALPS, ALP-like	IVIG, Steroids	Sirolimus	HSCT (reported)
IPEX	Steroids, Calcineurin inhibitors	Sirolimus	HSCT
IL-2RA, IL-2RB	Steroids, Infliximab	Sirolimus	HSCT
STAT3 GOF	Steroids, MMF	Ruxolitinib, Tocilizumab	HSCT
STAT1 GOF	Immunosuppression	Ruxolitinib, Baricitinib	HSCT
CTLA-4, LRBA, DEF-6	Steroids, MMF, Azathioprine	Sirolimus, Rituximab, Abatacept, Belatacept	HSCT (not reported for DEF-6)
IL-10, IL-10 receptor def	Steroids, Azathioprine	Infliximab, Anakinra	HSCT
APDS1, APDS2	IVIG, Steroids	Sirolimus, Rituximab, Leniolisib	HSCT
STAT3 LOF	Immunosuppression	Dupilumab, omalizumab	
STAT6 GOF	Immunosuppression	Ruxolitinib, dupilumab	
JAK1 GOF	Immunosuppression	Ruxolitinib, dupilumab	

For example, in IEI caused by hyperactivation of a specific protein, the use of small molecules to inhibit activity and return it to baseline levels has been studied for activated phosphoinositide 3-kinase-d syndrome (46).

Abatacept (CTLA-4 fusion protein, anti-CD-28); Anakinra (anti-IL-1 receptor); Baricitinib (JAK 1/2 inhibitor); Belatacept (CTLA-4 fusion protein); Infliximab (anti-TNF); IVIG (intravenous immunoglobulin); Leniolisib (PI3Kδ inhibitor); MMF (mycophenolate mofetil); Rituximab (anti-CD20); Ruxolitinib (JAK 1/2 inhibitor); Sirolimus (m-TOR inhibitor); Tocilizumab (anti-IL-6 receptor).

APDS is a very rare disease due to monogenic defects that cause constitutive T cell activation. It is an autosomal-dominant (AD) IEI caused by point mutations or deletions in one of the two genes encoding the two phosphoinositide 3-kinase δ (*PI3Kδ*) subunits. Heterozygous gain-of-PI3Kd-activity variants in PIK3CD or PIK3R1 cause APDS1 and APDS2, respectively, which show large phenotypic overlap (47, 48).

(49–51) APDS often presents early in life with an association of recurrent infections, lymphoproliferation, and autoimmune disease (i.e. autoimmune cytopenias, colitis, arthritis, and granulomatous skin lesions), deteriorating, later in life, in decrease lung function or lymphoma (52), with a higher risk in APDS2 patients.

Additional nonimmunological features such as neurodevelopmental delay or neuropsychiatric disorders, are more frequent in APDS-2 than APDS-1 patients (53, 54).

In APDS, both humoral and cell-mediated immune defenses are affected. Immunological characteristics that should lead to suspected APDS are represented by increased IgM serum levels with concomitant decreased to normal IgG and IgA levels, progressive B cell lymphopenia associated with a relative expansion of transitional B cells and plasmablasts, decrease of naïve T cell subsets with relative expansion of central and effector memory T cells (52, 55).

To date, there is no standard of care for treating APDS patients. Currently, the majority of treatments do not target the underlying pathogenesis of APDS and immune deficiency is treated with prophylactic antimicrobials and immunoglobulin replacement therapy (IRT), while immune dysregulation is generally treated with immunomodulatory therapies such as corticosteroids or the mTOR inhibitor rapamycin (52).

(52) Haematopoietic stem cell transplantation may represent a curative option. While HSCT has the potential to decrease the frequency of infections or severity of lymphoproliferation (56, 57), it may not correct non-immune/extra-haematopoietic manifestations.

Moreover, HSCT carries the risk of serious complications, including increased rates of graft rejection. In the literature, HSCT-related events represent the second most common cause of death in APDS patients (51).

As APDS is known to be caused by PI3Kδ overactivity, PI3Kδ inhibitors such as nemiralisib, seletalisib and leniolisib, have been studied as potential treatment options in limited patient populations.

In particular leniolisib, an oral systemic PI3Kδ inhibitor, has proven effective in inhibiting hyperactive PI3Kδ, as assessed by pAKT and pS6, normalizing PI3Kδ activity, improving immune dysregulation, and increasing patient well-being (58). The drug was noted to be well tolerated by patients with improved symptoms of lymphadenopathy, immune cell derangement and cytopenias. Moreover, it achieved reduction in lymph node and spleen volumes, normalized mean IgM levels, increased the number of naïve B cells and decreased the rate of transitional B cells (46).

In the absence of easy ways to measure cellular PI3K activity, it is difficult to know whether PI3Kδ activity is completely normalized. A commercially accessible method to monitor pAKT or other markers of pathway activity or inhibition would be helpful. The effects of leniolisib on pediatric populations requires further study.

For APDS, early onset predicts a severe disease course, calling for specific treatment studies in younger patients (54) and a pediatric trial is now recruiting (NCT05438407).

CTLA-4 insufficiency is another great example of when specific diagnosis can impact treatment as new therapies are introduced. CTLA-4 insufficiency is caused by loss-of-function mutations in CTLA-4, that impair the immunomodulatory function of regulatory T cells, causing immune activation (59). Affected individuals present with hypogammaglobulinemia, recurrent respiratory infections, autoimmunity, and lymphoproliferative complications (60). Therapeutic options for CTLA-4 insufficiency include IRT, antibiotic prophylaxis, while immune dysregulation is treated with immunosuppressive treatment, such as Rituximab, corticosteroids or mTor inhibitors. So far, only HSCT represents the long-term cure for CTL4-insufficiency, with high treatment-related morbidity and mortality (60). Abatacept, the soluble CTLA-4 Ig, is a targeted treatment option, replacing the missing or functionally impaired protein, and it is currently one of the most used therapeutic agents in these disease (61).

Abatacept is superior to immunosuppressants in controlling disease manifestations over the long term, especially when started early, and it may provide a safe and effective therapeutic alternative to transplantation (62).

## Conclusions

Patients with IEI are susceptible to developing a severe infection-related clinical phenotype, but the clinical consequences of this phenotype could represent the first sign in a significant percentage of patients. Early diagnosis of IEI is therefore essential to help identify suitable treatments that may delay or prevent irreversible organ damage (40).

The so-called “warning signs” of IEI (56) may help identify immunologic disorders, but unfortunately, these do not include signs of immune dysregulation.

Our purpose is to underline the need for early identification of common features of IEI and red flags to suspect IEI in the context of immune dysregulation for every patient. These new red flags, encompassing autoimmune cytopenias, chronic lymphoproliferation, cancer predisposition, allergies, endocrinopathies, enteropathy and vasculitis, should be considered as further warning signs to suspect a IEI (63).

In fact, identification of an underlying genetic diagnosis in immune dysregulation disorders is the prerequisite for disease-specific therapies, which are increasingly available for IEI (64).

The global initiative of the Jeffrey (65) Modell Foundation involving over 1,300 patients reported that genetic screening that interrogated a panel of 407 genes associated with IEI led to an alteration of clinical diagnosis and treatment in over 35% of patients (45).

Improvement in genetic testing has led to more specific diagnosis and delineation of immune dysregulation syndromes.

Currently, targeted therapies are available or show promise for the treatment of different IEIs.

## Footnotes

1 autoimmune lymphoproliferative syndrome; autoimmune polyendocrinopathy–candidiasis– ectodermal dystrophy; immune dysfunction, polyendocrinopathy, enteropathy, X-linked syndrome; and cytotoxic T lymphocyte–associated antigen 4 deficiency and gain-of- function mutations of signal transduce and activator of transcription 3.
